# Role of inflammation in the pathogenesis of hypertrophic cardiomyopathy: a T2-mapping CMR study

**DOI:** 10.1186/1532-429X-14-S1-P167

**Published:** 2012-02-01

**Authors:** Tevfik F Ismail, Niraj Mistry, Andrew Jabbour, Benjamin Hewins, Taigang He, Rick Wage, Ankur Gulati, Amy Mallorie, Dudley J Pennell, Sanjay K Prasad

**Affiliations:** 1 CMR Unit & NHLI Imperial College London, Royal Brompton Hospital & NHLI Imperial College London, London, UK

## Background

Hypertrophic cardiomyopathy (HCM) is associated with increased myocardial fibrosis and collagen deposition. Collagen reduces cardiovascular magnetic resonance (CMR) derived T2 times, however, the inflammatory milieu required for its deposition is often associated with localised oedema. Focal areas of high T2 signal intensity have been reported in association with areas of late gadolinium enhancement (LGE) in patients with HCM. However, this previous work has relied upon the subjective visual assessment of local changes in signal intensity. We hypothesised that quantitative CMR T2-mapping would reveal marked differences in myocardial T2 characteristics in patients with HCM relative to controls.

## Methods

Twenty-two consecutive patients with HCM (15 male, age 55.8±8.7 years) but no co-morbidity and 22 healthy controls (9 male, age 49.3±13.1 years) were studied. For each subject, a short axis mid-ventricular slice was acquired using a 1.5T Avanto (Siemens Healthcare, Erlangen, Germany). A breath-hold T2-prepared single-shot steady-state free precession (SSFP) sequence was used to produce three T2-weighted images with different T2 preparation times (0 ms, 24 ms, 55 ms). Parallel imaging was used to reduce the imaging time and motion-correction was applied to improve the accuracy of T2 measurement. T2 maps were produced by fitting the signal intensity-time exponential decay curve using a linear 2-parameter model after logarithmic transformation (CMRtools, Cardiovascular Imaging Solutions, London, UK). An inversion recovery prepared gradient echo sequence with the inversion time optimised to null normal myocardium was used 10 minutes after intravenous gadolinium contrast (Gadovist, Bayer-Schering, Berlin, Germany, 0.1 mmol/kg) to detect late enhancement. Imaging was repeated in an orthogonal phase encoding direction to exclude artifact.

## Results

There were no significant differences in baseline demographic characteristics between HCM and control patients. LV-wall thickness, indexed LV mass and ejection fraction were significantly higher in HCM patients (Table 1). In keeping with LV hypertrophy, the LV volumes were significantly smaller. None of the healthy controls exhibited any late enhancement whereas 18 (82%) HCM patients had areas of mid-wall enhancement, predominantly in the areas of hypertrophy. T2 times in patients with HCM were similar to controls with no evidence of any significant differences (HCM 54.1±2.5ms versus Controls 54.1±1.6ms, P=0.97).

**Table 1:** 

	Hypertrophic Cardiomyopathy	Controls	P Value
**N**	22	22	
**Male**	15	9	NS
**Age (years)**	55.8±8.7	49.3±13.1	NS
**LV-EDV (ml)**	138.6±33.0	141.2±32.0	NS
**LV ESV (ml)**	37.5±14.5	45.0±15.1	NS
**Indexed LV-EDV (g/m^2^)**	68.7±11.2	78.1±12.9	0.02
**Indexed LV-ESV (g/m^2^)**	18.5±6.4	24.7±6.8	0.004
**Ejection Fraction (%)**	73.5±6.1	68.8±5.5	0.01
**LV Mass**	185.9±68.2	105.0±30.8	<0.001
**Indexed LV Mass (g/m^2^)**	92.6±33.8	57.8±12.5	<0.001
**Maximum Wall Thickness (mm)**	18.8±4.9	7.8±1.2	<0.001

## Conclusions

Quantitative myocardial T2 mapping did not reveal any evidence of inflammation and was unable to detect the presence of any oedema in our cohort. Whilst this technique has been shown to be of utility in other cardiomyopathies such as dilated cardiomyopathy and Duchenne muscular dystrophy-associated cardiomyopathy, it does not appear to guide the evaluation of HCM.

## Funding

This work is supported by the NIHR Cardiovascular Biomedical Research Unit at the Royal Brompton and Harefield NHS Foundation Trust, and Imperial College. Dr Ismail is supported by the British Heart Foundation.

